# Psychopathological symptoms, defense mechanisms and time perspectives among subjects with alcohol dependence (AD) presenting different patterns of coping with stress

**DOI:** 10.7717/peerj.3576

**Published:** 2017-08-04

**Authors:** Katarzyna Iwanicka, Aneta Gerhant, Marcin Olajossy

**Affiliations:** 1 Addiction Treatment Clinic, Wolski Hospital, Warsaw, Poland; 2 Department of Psychiatry, Medical Academy in Lublin, Lublin, Poland

**Keywords:** Defense mechanisms, Patterns of coping with stress, Stress, Cluster analysis, Time perspectives, Psychopathological symptoms, Alcohol dependence

## Abstract

**Background:**

The problem of coping with stress is an important one in the context of development and persistence of alcohol dependence. In the literature to date very little attention has been paid to coping patterns construed as a configuration of specific coping styles, particularly as regards the functioning of addicted individuals. The aim of the study was to verify whether individuals with alcohol dependence characterized by different coping patterns differ with respect to the severity of psychopathological symptoms, defense mechanisms and time perspectives.

**Methods:**

Participants were given a battery of psychological tests—Coping Inventory for Stresfull Situations (CISS), Defense Style Questionnaire (DSQ 40), Syndrom Checklist (SCL-90) and Short Zimbardo Time Perspective Inventory (SZPTI-PL). The sample comprised 112 individuals with alcohol dependence, aged 20 to 63 years old, the average age was 37.86; 78 percent were men. There were identified three sub-groups of individuals characterized by a distinctive patterns of coping with stress —“emotional-avoidant”, “task oriented” and a “mixed one”.

**Results:**

Individuals with the predominant emotional-avoidant coping pattern are characterized by significantly higher severity of psychopathological symptoms, less mature defense mechanisms and past time perspectives. Subjects reliant on task-oriented coping pattern were characterized by the highest level of adaptation and the most constructive way of functioning in the face of difficulties.

**Conclusion:**

It is worth regarding the examination of patterns of coping as an indispensable element of collecting medical history from alcohol dependent individuals.

## Introduction

According to Lazarus & Folkmann (1994), coping with stress consists of cognitive and behavioral efforts to manage external or internal demands that are appraised as taxing, exceeding the resources of the person or endangering his or her welfare. A coping style is a relatively permanent repertoire of coping strategies specific to an individual. Endler & Parker (1990) distinguished between three basic stress coping styles: (1) a task-oriented style which involves taking problem-solving actions and plans, (2) an emotion-oriented style which concerns thoughts and actions aimed solely at reducing the tension caused by emotional stress, and (3) an avoidance-oriented style which is described as withdrawal from experiencing and engaging in solving a stressful situation. There are two avoidant coping subscales on the Coping Inventory for Stressful Situations (CISS) scale—distraction and social diversion. The first one involves engaging in substitute activities, which consists of redirecting attention to activities such as watching TV or reading a newspaper, while the second one involves seeking social interaction, which relates to the desire to obtain social support to reduce the tension. Campbell-Sills, Cohan & Stein (2006) indicated that both task-oriented coping and emotion-oriented coping contributed significantly to the prediction of resilience. It was also suggested (Aldao & Nolen-Hoeksema, 2012) that engagement in adaptive coping strategies negatively correlates with levels of psychopathological symptoms only when levels of maladaptive strategies are elevated.

The problem of coping with stress seems to be important in the context of the development and persistence of alcohol dependence (AD)^1^ 1As the study’s participants were assessed in accordance with ICD-10 alcohol dependence syndrome criteria, the term “alcohol dependence” will be used throughout the article; the equivalent of this term in the DSM 5 classification is “alcohol dependences (AD)”.and has long been raised in the literature on the subject (Opalach et al., 2016). Resorting to psychoactive substances in itself can be construed as an avoidant coping strategy (Woodhead et al., 2014; Mccormick et al., 1998). Holahan et al. (2001) indicated that both the emotion-oriented coping style and the avoidance-oriented style are strong predictors of AD. Further studies conducted among recovering AD individuals have shown that those who rely on the avoidance-oriented style, which manifests itself in shifting responsibility to others or dissociating from thinking about the difficulties in stressful situations, are more likely to resort to alcohol, which seems to be the main tension-reducing measure (Sinha, 2001). Developing alternative effective coping strategies seems to be crucial in the treatment of AD individuals. As shown by various studies, a change in the configuration of coping styles can occur as a result of therapeutic interventions carried out during a primary treatment program on an inpatient unit (Finney et al., 1998).

Cramer (2015) defines defense mechanisms as constructs acting as a counterforce against the push of the drives for discharge. Individuals using more adaptive coping styles, such as task-oriented coping, are characterized by greater ego strength and the use of more mature defense mechanisms than those presenting less adaptive styles such as emotional and avoidant oriented (Moos & Halogen, 2003). Studies have shown that AD is associated with more frequent use of immature defense mechanisms such as pseudo-altruism, autistic fantasy, acting out and isolation. Studies have shown that AD individuals use immature and neurotic defense mechanisms such as pseudo-altruism, autistic fantasy, acting out, isolation, projection, splitting or somatization more frequently than healthy people (Taskent et al., 2011; Evren et al., 2012a). At the same time, a positive correlation between the tendency to use immature defense mechanisms and the extent of AD and a tendency to antisocial behavior was observed (Taskent et al., 2011). Bagheri, Azadfallah & Ashtiany (2013) also found that AD women score significantly higher than healthy women with respect to defense mechanisms such as acting out or autistic fantasy, while achieving lower scores for mature defense mechanisms such as sublimation. In young people with AD a stronger manifestation of the acting out mechanism is associated with a higher risk of self-injury, while less frequent use of anticipation, classified as a mature defense mechanism, is a predictor of suicidal behavior (Evren et al., 2012b).

Coping strategies tend to be perceived as mature, voluntary and intentional whereas defense mechanisms are considered to be unconscious, involuntary, rigid and automatic (Diehl et al., 2014; Crasovan, 2013). Furthermore, coping styles contrary to defense mechanism, which are oriented towards internal conflicts, are crucial to positive adaptation to external reality (Crasovan, 2013). However, empirical data suggest that these differences are much more blurred than in theoretical models (Kramer, 2010). Callahan & Chabrol (2004) proposed a sequential model where defense mechanism precede coping processes. According to that theory, defense mechanism and coping strategies are different psychological constructs which are functionally linked. Defense mechanisms influence reality perception and create threat representations, secondarily affecting coping strategies. Thus, adaptive coping mechanisms may be preceded by non-adaptive defense mechanisms or vice versa (Kramer, 2010). That model has important implications in terms of therapeutic approach and directs attention to analysis of defense mechanisms as an indispensable step in improvement of coping resources (Kramer, 2010; Crasovan, 2013). Therefore, assessment of defense mechanisms in relation to preferred stress coping patterns in AD individuals seems to be interesting and helpful in therapy.

Mercier et al. (1992) indicated that AD individuals presented significantly higher severity of psychopathological symptoms, especially psychoticism (reflecting the continuum of behaviors from mild social withdrawal to the first rank symptoms of psychosis) than healthy individuals. AD individuals often report various somatic ailments such as headaches, joint pain, a burning sensation in the chest, weakness and difficulty in breathing (Hasin & Katz, 2007; Tien, Schlaepfer & Fisch, 1998). When compared to the control group, AD individuals are characterized by more severe depressive symptoms (Skule et al., 2014; Gamble et al., 2010; Strowig, 2000; Allen et al., 1990). It has been suggested that a high severity of psychopathological symptoms, particularly of anxiety and depression in AD individuals, is associated with a higher risk of relapse (Driessen et al., 2001). AD individuals, compared with the control group, obtain significantly higher scores on the psychoticism scale (Chadhury, Das & Ukil, 2006). Moreover, AD individuals more frequently experience hostility, irritability, and aggression (both verbal and nonverbal) in comparison with the healthy population (Ilyuk et al., 2012). Endler, Parker & Butcher (2003) found a positive association between an emotion-oriented coping style and various measures of psychopathology in the MMPI-2 scale, including depression, anxiety, obsessiveness, anger, and low self-esteem. Task-oriented coping-styles were unrelated to scores on these measures.

The way people experience their past and plan their future influences their behavior and their choice of the coping strategies (Boltova & Hachaturova, 2013). Time perspective can be defined as an often unconscious personal attitude that every individual manifests towards time (Zimbardo & Boyd, 2008). It is also the process by which the continuum of life is divided into categories of time to help give individuals’ lives order, cohesion and importance. Zimbardo & Boyd (2008) identified five time perspectives. The first of them, past negative, characterizes individuals who may misremember the past in a negative way. They might experience higher levels of anxiety and depression, as well as rumination. People with a past positive time perspective concentrate on positive aspects of their life history. They tend to be more nostalgic and bound to rituals. The third time perspective, present fatalism, characterizes individuals who believe they are powerless and that they can not influence their future. People presenting present hedonism, the fourth time perspective, strive to maximize perceived pleasure. They can be characterized by a high level of novelty and sensation seeking. The last but not least time perspective is a future one, associated with the representation of future states and organising the individual’s activity around life goals. Keough, Zimbardo & Boyd (1999) indicated that there is a negative correlation between a future time perspective and reported substance use, but a positive correlation between present time perspective and reported substance use. As shown by the studies, more frequent reliance on action-oriented coping strategies is accompanied by a heightened future time perspective. By contrast, those with high scores on the present time perspective scale frequently use maladaptive strategies such as a focus on avoidance, collapsing into helplessness or experiencing difficult emotions such as anger (Wills, Sandy & Yaeger, 2014). It has, however, been suggested that participation in rehabilitation treatment may entail a change in time perspective as well as an orientation toward the future (Alvos,Greyson & Ross, 1993). Furthermore, a future time perspective is a strong predictor of abstinence upon completion of the treatment program (Lennings, 1996). Beenstock, Adams & White (2011) indicated that individuals with greater future oriented time perspective are less prone to addictive health behaviors like alcohol abuse, since they rather concentrate on long term negative effects of drinking then short term positive outcomes. Time perspective, as a fairly stable construct, has an impact on the action strategies taken by individuals. Boniwell & Zimbardo (2004) showed that in a conflict situation, future and past oriented people are more prone to cooperate than present oriented ones. It seems important to examine the relationship between time perspectives and coping styles among AD individuals, who in this study find themselves in specific circumstances of hospitalization at the addiction treatment ward.

In the literature to date some researchers’ attention has been focused on coping patterns (called sometimes “profiles”), described as a configuration of specific coping styles, in the functioning of AD individuals (Roos & Witkiewitz, 2016). However, most research on coping styles and their correlates has focused on healthy populations (Doron et al., 2015; Eisenbarth, 2012; Wijndaele et al., 2007) or somatically ill patients (Dunkel-Schetter et al., 1992; Smith & Wallston, 1996; Losiak, 2001). A key question to be answered by our study is which patterns of coping with stress can be distinguished among individuals with alcohol dependence. Moreover, the study presented in this paper is intended to complement existing research on the functioning of individuals with alcohol dependence presenting different patterns of coping in terms of the severity of their psychopathological symptoms, defense mechanisms and time perspectives. In light of studies mentioned in the introduction, it can be concluded that variables such as severity of psychopathological symptoms, defense mechanisms and time perspective seem to be strong indicators of psychological well-being and are strongly connected with a higher risk of relapse among alcohol dependent individuals.

## Materials and Methods

### Participants

A total of 112 AD individuals (*N* = 91 males, *M* = 38; *SD* = 20.87) took part in the study. They were participants in an 8-week abstinence-based inpatient treatment program the research was conducted within first two weeks of the treatment. The diagnosis of alcohol dependence was based on the ICD-10 classification criteria (World Health Organization, 2007) and a psychiatric assessment (structured interview based on the ADIT questionnaire and list of other questions allowing carrying out a differential diagnosis). The conditions for inclusion in the study were as follows: the participant had to (1) be at least 18 years of age (2) sign an informed consent form and (3) have abstained from alcohol for at least two weeks. Since with this length of abstinence withdrawal symptoms did not occur among participants, no psychopharmacological treatments were given. The study was approved by the Committee on Bioethics of the Medical University of Lublin (No. KE-0254/145/2011) and was carried out in accordance with the Committee’s recommendations. Each of the participants was informed that the data obtained would be kept confidential. Completion of the survey was voluntary. Participants were briefed as to the purpose and procedures of the study.

### Instruments

The present study was a correlational study. Four psychological tests were used to measure variables such as stress coping styles, defense mechanisms, psychopathological symptoms and time perspectives—the Coping Inventory for Stressful Situations (CISS), the Defense Style Questionnaire (DSQ 40), the Symptom Checklist (SCL-90) and the Short Zimbardo Time Perspective Inventory (SZPTI-PL). Additionally, a personal questionnaire designed by the authors was applied to measure variables such as: years of education, place of residence, the age of alcohol use initiation, alcohol dependence in the family and attendance at the AA meetings.

**The Coping Inventory for Stressful Situations (CISS)** (Endler & Parker, 1994) adapted into Polish by Strelau et al. (2005) was used. The tool consists of three scales: the emotion-oriented style (EOS), the task-oriented style (TOS) and the avoidance-oriented style (AOS). Each of the subscales contains 16 items. Moreover, the last scale, avoidant coping, has two subscales—distraction and social diversion. Respondents indicate the frequency of a given activity on a scale of 1 to 5. The Polish adaptation is characterized by high internal reliability, as the Cronbach’s alpha reliability coefficient for the individual scales lies within the range of 0.73 (SSI scale) to 0.80 (AOS scale). Moreover, the scales are relatively independent, as confirmed by a low correlation between them (0.08–0.13).

**The Defense Style Questionnaire (DSQ 40)** developed by Andrews, Singh & Bond (1993) was used. The Polish adaptation was developed by Bogutyn et al. (1999). In this questionnaire the authors distinguished 20 defense mechanisms, each of which belonged to a group of mature, neurotic or immature mechanisms. Each mechanism is represented in the questionnaire by two items. Listed among mature ones are sublimation, humor, anticipation and suppression. Neurotic mechanisms include undoing, pseudo-altruism, idealization and reaction formation. Immature mechanisms include projection, passive aggression, acting out, isolation, devaluation, autistic fantasy, denial, displacement, dissociation, splitting, rationalization and somatization. Participants were asked to indicate their degree of agreement or disagreement with each statement on a 9-point scale (from 1—“strong disagreement” to 9—“strong agreement”). The original version of the questionnaire is characterized by satisfactory reliability and validity (Andrews, Singh & Bond, 1993). **The** **Symptom Checklist (SCL-90)** by Derogatis, Lipman & Covi (1973), as adapted into Polish by Brodziak (1981), was administered to measure the severity of psychopathological symptoms. The checklist comprises 90 statements assigned to nine groups of clinical symptoms. These include: somatization, compulsion, interpersonal sensitivity, anxiety, depression, hostility, phobias, paranoid thinking, and psychoticism. Each of the nine symptom dimensions is made up of 6 to 13 items. Respondents circle the severity of symptoms on a scale from 0 (“not at all”) to 4 (“extremely”), where a high score indicates a high severity of psychopathological symptoms. They are asked to indicate how much that problem has bothered or distressed them during the past four weeks up to the day of the test.

**The Short Zimbardo Time Perspective Inventory (SZPTI-PL)** (Zimbardo & Boyd, 1999), as adapted into Polish by Cybis et al. (2012). The authors identified five time perspectives: past-negative, past-positive, present hedonism, present fatalism and future. Each element comprises three questionnaire items. For each item, the subject determines to what extent he or she agrees with the statement on a 5-point scale (1—totally disagree, 5—completely agree). Reliability was tested and proved to be satisfactory (Zhang, Howell & Bowerman, 2013).

### Statistical analysis

The scores were statistically analyzed using STATISTICA 10.0 PL. In order to identify stress coping patterns, a nonhierarchical cluster analysis was performed. This divided the group of individuals into subgroups obtaining similar scores on factors identified by the CISS. Cluster analysis results in grouping objects based on their mathematically determined similarity. In each of the identified subgroups statistical distribution of the data was checked using the Kolmogorov Smirnov test with Lilliefors correction and the Shapiro Wilk test. Since the sample data had a normal distribution, parametric tests were applied. Next, to test the differences in the intensity of the psychopathological symptoms, defense mechanisms and time perspectives in the three clusters one-way analysis of variance (ANOVA) was used. A Tukey post hoc test was applied to check level of statistical significance of obtained differences .

## Results

Out of the group of 112 alcohol dependent individuals three clusters were identified showing a distinctive distribution of CISS scores on the specified scales. Statistically significant differences in relation to coping styles were observed between the 3 clusters of individuals with alcohol dependence (Table 1). Further analysis using the Tukey post hoc test showed that the significant differences were revealed between clusters I and III. No statistically significant difference was observed between clusters II and III (Table 1).

**Table 1:** Differences between coping styles in the three clusters.

CISS scales	Cluster I *n* = 40		Cluster II *n* = 32		Cluster III *n* = 33		ANOVA		Cluster I–II	Cluster I–III	Cluster II–III
	*M*	*SD*	*M*	*SD*	*M*	*SD*	*F*	*p*	*p*	*p*	*p*
TOS	59.44	59.44	43.97	6.02	57.70	5.91	84.36	**.001**	.001	.001	ns
EOS	55.02	55.02	47.88	7.74	43.21	9.03	20.29	**.001**	.001	.001	.001
AOS	51.27	51.27	45.00	6.16	34.65	6.92	67.80	**.001**	ns	.001	.001
D	23.82	23.82	22.21	3.60	13.12	2.71	84.10	**.001**	.003	.001	ns
SD	17.04	17.04	13.88	3.65	14.00	4.63	7.70	**.001**	<.001	.004	<.001

TOS—task-oriented style, EOS—emotion-oriented style, AOS—avoidance-oriented style, D—distraction, SD—social diversion.

**Figure 1:**
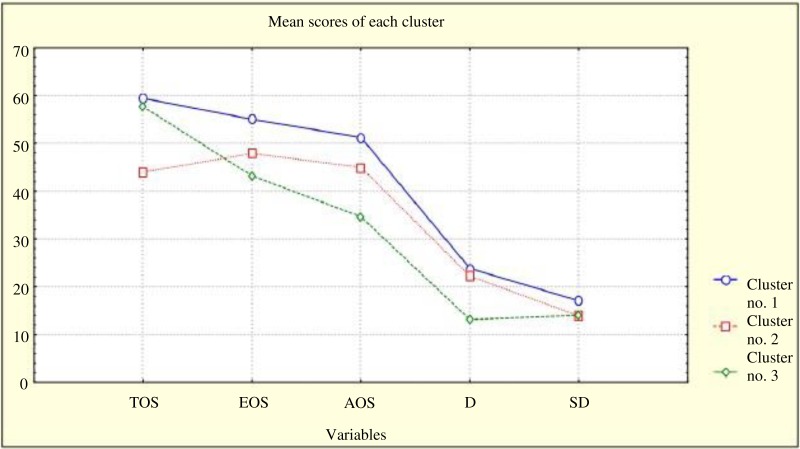
Differences among individuals from particular clusters with respect to coping styles. TOS, task-oriented style; EOS, emotion-oriented style; AOS, avoidance-oriented style; D, distraction; SD, social diversion.

Group I included forty-five individuals (*N* = 35 males; *M* age = 39.16; *SD* = 10.4), group II was composed of thirty-three individuals (*N* = 29 males; *M* age = 34.49; *SD* = 12.91) and group III was consisted of thirty-four participants (*N* = 27 males; *M* age = 41.97; *SD* = 10.84). A chi-square test with Bonferroni correction (Stanisz, 2007) was conducted and no between group difference was detected with respect to age (*χ*^2^(2) = 1.2, *p* = .55), sex (*χ*^2^(2) = 1.38, *p* = .50), years of education (*χ*^2^(6) = 14.89, *p* = .21), place of residence (*χ*^2^(6) = 5.36, *p* = .5), age of alcohol use initiation (*χ*^2^(2) = 1.2, *p* = .38), alcohol dependence in the family (*χ*^2^(2) = 0.29, *p* = .86) or attendance at AA meetings (*χ*^2^(2) = 3.94, *p* = .14). The division into three groups is justified by statistical and substantive arguments: the number of subgroups that enables their comparison with specific variables and a clear psychological significance of identified types of coping styles (Fig. 1).

The first pattern of coping with stress (cluster I) was designated as “**emotional-avoidant coping**,” as individuals in this cluster relied on the emotion- and avoidance-oriented coping styles more frequently than other individuals. The pattern presented by the individuals in the second cluster (cluster II) was designated as “**mixed coping**” as the individuals did not prefer one particular coping style and used all with similar frequency. The third and last of these patterns (cluster III) was characterized as “**task-oriented coping**” as the individuals classified in this cluster focused on planning and applying solutions to a specific problem, while rarely relying on other styles. Individual groups varied in terms of defense mechanisms, the severity of psychopathological symptoms and time perspectives.

With a one-way analysis of variance (ANOVA), the intensity of the psychopathological symptoms presented in the three clusters was compared (Table 2). Further analysis using the Tukey post hoc test showed the level of significance of differences between the three clusters of alcohol-dependent individuals in the intensity of their psychopathological symptoms (Table 2).

**Table 2:** Differences between intensity of psychopathological symptoms in the three clusters.

SCL-90 scales	Cluster I Emotional-avoidant		Cluster II Mixed coping		Cluster III Task oriented		ANOVA		Clus. I–I	Clus. I–III	Clus. II–III
	*M*	*SD*	*M*	*SD*	*M*	*SD*	*F*	*p*	*p*	*p*	*p*
Somatization	15.70	9.77	10.21	7.35	9.39	8.14	6.18	**.003**	0.02	.01	n.i.
Obsessive- compulsive	19.05	6.55	16.30	5.43	13.33	6.57	7.84	**.001**	ns	.01	ns
Interpersonal sensitivity	14.47	7.10	12.39	6.87	9.67	7.45	4.22	**.017**	ns	.01	ns
Depression	22.91	9.03	19.06	8.57	15.42	10.05	6.20	**.003**	ns	.01	ns
Anxiety	17.16	7.63	12.64	6.64	10.67	7.82	7.79	**.001**	.02	.01	ns
Hostility	7.42	4.27	7.48	5.19	4.85	4.22	3.75	**.027**	ns	.05	.05
Phobic anxiety	6.28	5.16	5.61	5.57	3.39	4.84	3.02	**.053**	ns	.05	ns
Paranoid ideation	10.95	4.18	10.03	7.40	8.27	4.79	2.23	.112	–	-	–
Psychoticism	11.65	6.09	9.58	5.42	6.12	4.35	9.80	**.001**	ns	.01	.03
Poor appetite	1.33	1.17	1.06	1.12	0.97	1.26	0.95	.392	–	–	–
Overeating	1.19	1.10	2.39	7.55	0.76	1.06	1.33	.268	–	–	–
Trouble falling asleep	2.21	1.41	1.70	1.29	1.36	1.54	3.46	**.035**	ns	.03	ns
Awaking in the early morning	2.23	1.25	1.88	1.41	1.61	1.41	2.05	.133	–	–	–
Restless/interrupted sleep	2.09	1.23	1.58	1.23	1.36	1.41	3.28	**.041**	ns	.04	ns
Thoughts of death / dying	0.93	1.22	1.70	5.73	0.61	1.09	0.96	.385	–	–	–
Feeling of guilt	2.53	1.28	2.27	1.15	1.67	1.24	4.75	**.011**	ns	.01	ns

A significant difference on the SCL-90 scales was found between the emotional-avoidant and task-oriented coping clusters (I and III). No significant difference was observed between the mixed coping and task-oriented clusters (II and III) (Table 2). Individuals with the predominant emotional-avoidant coping pattern are characterized by a significantly higher severity of psychopathological symptoms such as somatization (*p* = .01), obsessive-compulsive behaviors (*p* = .01), interpersonal sensitivity (*p* = .01), depression (*p* = .01), anxiety (*p* = .01), hostility (*p* = .05), phobic anxiety (*p* = .05) psychoticism (*p* = .01), trouble falling asleep (*p* = .03), restless or interrupted sleep (*p* = .04) and a feeling of guilt (*p* = .01), compared to those presenting the task-oriented pattern (Table 2). The participants in the emotional-avoidant cluster I differed from those in the mixed one, as they experienced significantly more severe anxiety (*p* = .02) and somatization symptoms (*p* = .02) (Table 2). Moreover, the individuals in the task-oriented coping cluster achieved significantly lower scores in terms of hostility (*p* = .05) and psychoticism (*p* = .03) than individuals in cluster II—the mixed one (Table 2).

The intensity of defense mechanisms was checked with a one-way analysis of variance (ANOVA) (Table 3). The level of significance of differences between the three identified clusters of individuals with alcohol dependence with respect to defense mechanisms was measured with the Tukey pos hoc test (Table 3).

**Table 3:** Differences between intensity of defense mechanisms in the three clusters.

DSQ 40 scales	Cluster I Emotional-avoidant		Cluster II Mixed coping		Cluster III Task oriented		ANOVA		Cluster I–II	Cluster I–III	Cluster II–III
	*M*	*SD*	*M*	*SD*	*M*	*SD*	*F*	*p*	*p*	*p*	*p*
Sublimation	**11.51**	**3.87**	**8.68**	**3.23**	**11.10**	**3.28**	**6.31**	**.003**	.003	ns	.02
Humor	11.22	3.73	11.03	4.05	10.94	3.58	0.05	.948	–	–	–
Anticipation	11.46	3.10	10.00	3.44	10.68	3.70	1.67	.194	–	–	–
Suppression	10.41	3.07	10.35	4.29	10.00	3.21	0.13	.874	–	–	–
Undoing	10.66	3.79	9.97	3.71	9.81	3.18	0.58	.559	–	-	–
Pseudo-altruism	11.66	3.57	10.55	4.23	10.55	3.25	1.11	.332	–	–	–
Idealization	8.63	4.81	7.94	4.41	6.35	3.72	2.43	.093	–	-	–
Reaction formation	10.27	3.67	9.10	4.15	8.87	3.05	1.56	.216	–	-	–
Projection	**9.32**	**4.02**	**7.74**	**4.15**	**6.45**	**3.60**	**4.75**	**.011**	ns	.01	ns
Passive aggression	**8.80**	**4.30**	**8.68**	**4.09**	**6.13**	**3.41**	**4.68**	**.011**	ns	.02	.04
Acting out	10.07	4.94	10.10	4.94	9.35	3.92	0.27	.768	–	–	–
Isolation	9.51	3.65	10.32	4.56	9.00	4.31	0.81	.449	–	–	–
Devaluation	8.37	3.29	7.03	3.44	7.16	3.45	1.75	.179	–	–	–
Autistic fantasy	8.80	4.63	9.16	4.69	6.81	4.69	2.35	.100	–	-	–
Denial	7.34	2.60	7.90	4.09	6.29	3.67	1.77	.175	–	–	–
Displacement	**8.29**	**3.81**	**7.10**	**4.27**	**5.42**	**3.40**	**4.94**	**.009**	ns	.01	ns
Dissociation	7.95	3.60	7.74	3.90	6.16	3.41	2.40	.096	–	–	–
Splitting	**10.49**	**3.84**	**8.97**	**4.76**	**7.65**	**3.58**	**4.37**	**.015**	ns	.01	ns
Rationalization	11.59	3.22	11.06	4.42	11.16	3.25	0.21	.807	–	–	–
Somatization	9.68	3.88	9.13	4.18	8.68	3.72	0.59	.557	–	–	–

Significant differences on the DSQ 40 scales were identified between cluster I and cluster III (Table 3). Individuals in the emotional-avoidant cluster (I) achieved significantly higher scores than those in the task-oriented cluster (III) in relation to the intensity of their use of immature defense mechanisms such as projection (*p* = .01), passive aggression ( *p* = .02), displacement (*p* = .01) and splitting (*p* = .01). At the same time, individuals in clusters I and III used a mature mechanism, i.e., sublimation (*p* = .03), significantly more frequently than those in cluster II. Participants in the mixed coping cluster (II) are the least likely, however, to use a defense mechanism such as passive aggression (*p* = .04), compared to the individuals in the other two clusters (Table 3).

With a one-way analysis of variance (ANOVA), the intensity of the presented time perspectives of the three clusters was compared. It has also been suggested that AD individuals with different coping patterns differ in terms of time perspectives (Table 4). The Tukey post hoc test indicated statistically significant differences exist between individuals in emotional-avoidant (I) and task oriented (III) clusters and individuals in mixed (II) and task oriented (III) coping clusters (Table 4). The participants in the emotional-avoidant cluster are characterized by significantly greater focus on the past perspective, both positive (*p* = .04) and negative (*p* = .01), compared to the individuals in the task-oriented cluster. Individuals in the task oriented (III) cluster are characterized by a greater focus on the future than those in mixed (II) cluster (*p* = .001), which may be associated with greater ease of determining goals and pursuing them and focusing on future events (Table 4).

**Table 4:** Differences between time perspective in the three clusters.

Time perspectives	Cluster I Emotional-avoidant		Cluster II Mixed coping		Cluster III Task oriented		ANOVA		Clus. I–II	Clus. I–III	Clus. II–III
	*M*	*SD*	*M*	*SD*	*M*	*SD*	*F*	*p*	*p*	*p*	*p*
Past-negative	11.36	2.34	9.94	3.15	9.21	3.62	5.23	**.007**	ns	.01	ns
Past-positive	11.62	2.90	11.36	3.00	9.91	3.09	3.47	**.035**	ns	.04	ns
Present- fatalism	8.82	2.58	10.91	10.23	7.18	2.61	3.30	**.040**	ns	ns	ns
Present- Hedonism	8.84	2.22	9.55	2.81	8.38	2.83	1.71	.185	–	–	–
Future	11.09	2.46	9.85	2.49	11.97	2.24	6.60	**.002**	ns	ns	.001

## Discussion

The aim of the study was to verify whether individuals with alcohol dependence characterized by different coping patterns differ with respect to the severity of their psychopathological symptoms, defense mechanisms and time perspectives.

The results of our study have shown that alcohol dependent individuals presenting the **emotional-avoidant coping pattern**, compared to those characterized by the task-oriented pattern, suffer from a greater severity of psychopathological symptoms. Those findings correspond with the results of other authors, who reported that the preference for emotional coping is associated with more severe depressive symptoms (Billings & Moos, 1984) anxiety, compulsive thoughts and hostility (Endler, Parker & Butcher, 2003). It has also been shown that strategies involving avoidance of thinking about the problem and denying the difficulties is connected with anxiety symptoms and a paradoxical increase in intrusiveness of repressed thoughts (Wegner & Zanakos, 1994; Wenzlaff & Wegner, 2000). Similarly, other authors have indicated the existence of a positive relationship between the avoidance-oriented style and greater severity of depressive symptoms (Cronkite et al., 1998; Sherbourne, Hays & Wells, 1995). On the other hand, it has been shown that individuals with substance use disorders characterized by a high level of aggression and hostility often cope with stress by distancing themselves and avoiding stimuli (McCormick & Smith, 1995). Based on the data obtained in this study, one can conclude that in alcohol dependent individuals the use of avoidant and emotion-oriented coping strategies was associated with a higher level of anxiety, depression and psychoticism. For such individuals, further evaluation for comorbid mental disorders and the possible introduction of pharmacological treatment is worth considering (Scott, Gilvarry & Farrell, 1998). Yet Hall & Farrell (1997) postulate a more practical solution involving the education of all personnel working with alcohol dependent individuals on the psychopathological symptoms most commonly comorbid with alcohol dependence.

It has also been shown in this paper that reliance on the emotional-avoidant coping pattern in AD individuals, compared to those who mainly use task-oriented strategies, is associated with a greater tendency to use immature defense mechanisms such as passive aggression, projection, denial and splitting. According to Sala et al. (2015), mature defense mechanisms are accompanied by more adaptive strategies of coping with stress such as positive reevaluation or developing a further plan of action. An interesting result obtained in our study is that contrary to reports in the literature (Ward & Rothaus, 1991), the cluster of individuals presenting an emotional-avoidant coping pattern did not differ in terms of the intensity of reliance on the denial mechanism but more frequently applied displacement and splitting.

The results showed that in individuals relying on emotional-avoidant coping, compared to those who rely on task-oriented coping, time perspective was significantly more often oriented toward the positive or negative past than the present or future. Zimbardo & Boyd (2008) and Wills, Sandy & Yaeger (2014) emphasized that future-oriented individuals present more constructive, action-oriented coping strategies. That was also observed in our study, where alcohol-dependent individuals with the task-oriented pattern of coping preferred the future time perspective to the greatest extent.

Findings obtained in the study indicated that the emotional-avoidant coping pattern, due to the configuration of the non-adaptive coping styles, immature defense mechanisms, severe psychopathological symptoms and past time perspectives is not only a predisposing factor to alcohol dependence, but it also poses a risk of relapse upon completion of treatment (Moos & Moos, 2006). It would be useful to monitor the patients upon completion of treatment to verify what percentage stays abstinent, as well as whether the configuration of coping styles has changed along with defense mechanisms, psychopathological symptoms and time perspectives. Thus, individuals presenting the emotional-avoidant coping pattern require increased concern from therapeutic staff due to the unfavorable prognosis as to the development of alcohol dependence. One should also keep in mind the increased risk of comorbidity of other psychiatric disorders such as anxiety disorders, which can contribute to relapse among these patients (Kushner, Abrams & Borchardt, 2000).

Task-oriented coping is associated with less severe psychopathological symptoms. Individuals relying on task-oriented coping are less prone to hostility in interpersonal relations, suspicion and grandiose delusions than individuals following the other patterns identified. They also experience anxiety, depressed moods, compulsions, phobias and sleeping difficulties significantly less frequently than alcohol dependent individuals with emotional-avoidant coping. They show a significantly lower severity of immature defense mechanisms such as projection, denial and splitting when compared to the individuals in the first cluster, and use passive aggression less often than individuals relying on emotional-avoidant coping and mixed coping. Alcohol dependent individuals relying on task-oriented coping use mature defense mechanisms such as sublimation more frequently than those presenting mixed coping. This result is interesting due to the possible use of a mature defense mechanism, i.e., sublimation, in such individuals undergoing alternative therapies such as art therapy and creative workshops. Horay (2006) shows that these forms of therapeutic influence can be effective support for alcohol dependent individuals, as they help facilitate the development of a constructive vision of life in sobriety.

The conclusion that can be drawn based on the results of our study is that presenting the task-oriented coping pattern is correlated with future time perspective among participants with AD. Having adopted such a time perspective, they are focused on success and meeting the commitments made. They are more likely to delay gratification, but it should be remembered that in this cluster of participants this ability is lower than in the general population. Zimbardo & Boyd (2008) argued that future-oriented individuals are less depressive and anxious, since they dwell on past events less often and do not focus on losses, but instead on challenges.

Participants presenting the mixed coping pattern relied equally frequently on all coping styles. However, in comparison to the other two clusters, their use of the task-oriented style is the least frequent and they are the least likely to use sublimation as a mature defense mechanism. However, they use the mechanism of passive aggression more often than the individuals in the third cluster. This shows that individuals relying on this coping pattern face greater difficulties in planning their activities or setting their goals in life, and find it harder to delay gratification for their actions.

## Limitations

This study has several limitations. Firstly, in the sample of individuals, men were over- represented compared to women. Previous studies have shown that women addicted to alcohol differ from men in terms of intensity of coping styles they use (Timko, Finney & Moos, 2005; Cooper et al., 1992) and thus it may also be reflected in coping patterns, or a configuration of a number of styles. Secondly, the group covered by the study was relatively small and its selection was not random. Lastly, the study is cross-sectional and depicts only static relationships between examined variables.

## Conclusions

The findings of this study may have a practical application in therapeutic work with with alcohol dependent individuals. The participants with an emotional-avoidant stress coping pattern are characterized by greater severity of psychopathological symptoms, more immature defense mechanisms and concentration on the past compared to those with a task-oriented pattern. An interesting issue to explore in the future is how a change in stress coping patterns influence defense mechanisms and time perspectives in alcohol dependent individuals. The studies presented in this paper partly fill the gap in research on the relationship between particular patterns of coping with stress, psychopathological symptoms, defense mechanisms and time perspectives among AD. Further studies are needed to verify how individuals presenting different patterns of coping with stress will benefit from the therapeutic programme and what kind of proposed treatments would be most favorable for them.

##  Supplemental Information

10.7717/peerj.3576/supp-1 Data S1 Raw data

## Additional Information and Declarations

### Data Availability

The following information was supplied regarding data availability: The raw data has been supplied as Data S1.
